# *Clostridium perfringens* panophthalmitis and orbital cellulitis: a case report

**DOI:** 10.1186/s12886-018-0751-0

**Published:** 2018-04-10

**Authors:** Ghita Guedira, Nabil Taright, Hélène Blin, Thameur Fattoum, Jordan Leroy, Youssef El Samad, Solange Milazzo, Farida Hamdad

**Affiliations:** 1 Ophthalmology Department, Amiens-Picardie University Hospital, Amiens, France; 2 Ophthalmology Department, Beauvais Hospital, Beauvais, France; 3 Clinical Bacteriology Department, Amiens-Picardie University Hospital, Amiens, France; 4 Infectious diseases Department, Amiens-Picardie University Hospital, Amiens, France; 5 Centre de Biologie Humaine, CHU Amiens-Picardie, Avenue R. Laennec, 80054 Amiens Cedex1, France

**Keywords:** *C. perfringens*, Panophthalmitis, Diagnosis, Treatment

## Abstract

**Background:**

*Clostridium perfringens* is an uncommon pathogen in endophthalmitis, causing rapid destruction of ocular tissues. *Clostridium perfringens* infection typically occurs after penetrating injury with soil-contaminated foreign bodies.

**Case report:**

Here, we describe the case of a 17-year-old male who sustained a penetrating injury with a metallic intraocular foreign body and who rapidly developed severe *C. perfringens* panophthalmitis with orbital cellulitis. He was managed by systemic and intravitreal antibiotics, resulting in preservation of the globe, but a poor visual outcome.

**Conclusion:**

Clostridial endophthalmitis secondary to penetrating injuries is a fulminant infection, almost always resulting in loss of the globe in the case of advanced infection. When feasible, early vitrectomy and intravitreal antibiotics should be considered in patients with penetrating eye injuries with contaminated foreign bodies.

## Background

Post-traumatic infectious endophthalmitis is an uncommon but severe complication of ocular trauma. The outcome of the infection varies according to the type of injury, the microorganisms involved and the time between injury and treatment [1]. The visual prognosis is also determined by the anatomic site of the intraocular foreign body (IOFB), as the majority of IOFBs are located in the posterior segment of the eye, which is associated with a poorer visual prognosis than those located in the anterior segment [2–4].

Clinical signs of endophthalmitis include severe pain, purulent exudate from the site of injury, eyelid edema, chemosis, and hypopyon. The infection can progress, resulting in panophthalmitis and can be complicated by possibly life-threatening orbital cellulitis.

Clostridial endophthalmitis secondary to penetrating injuries is a fulminant infection, almost always associated with loss of the eye [5], and antibiotics are unlikely to prevent this process.

## Case report

A 17-year-old male was admitted to the Ophthalmology Department of Amiens-Picardie University Hospital on May 28, 2013. He described a 24-h history of loss of vision and pain after projection of a foreign metallic into the left eye following a perforating eye injury while performing housework. He did not present any notable medical history.

**Fig. 1:**
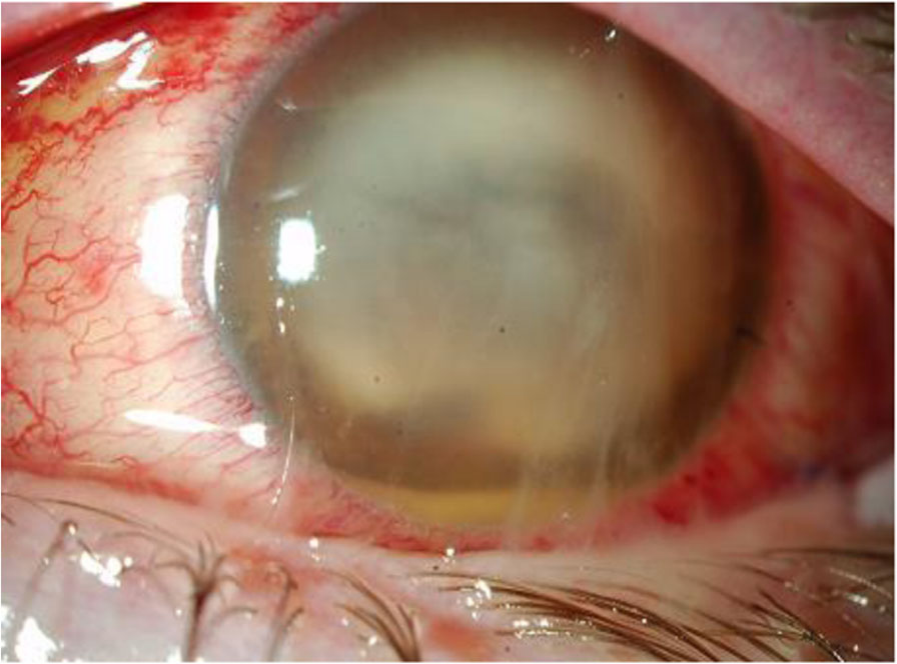
The conjunctiva is congested with subconjunctival hemorrhage

The patient presented elevated intraocular pressure and palpebral edema associated with pre septal cellulitis. Slit lamp examination revealed extensive chemosis with fibrin in the anterior chamber and hypopyon. The cornea was whitened and the iris and crystalline lens were not visible [Fig. 1].

Vision was impaired to no light perception (NLP). All eye movements were grossly restricted, with mild proptosis and swollen lids. The patient was afebrile, but presented an elevated white blood cell count of 30,200 cells/mm^3^ and C-Reactive Protein (CRP) of 52.9 mg/L.

**Fig. 2:**
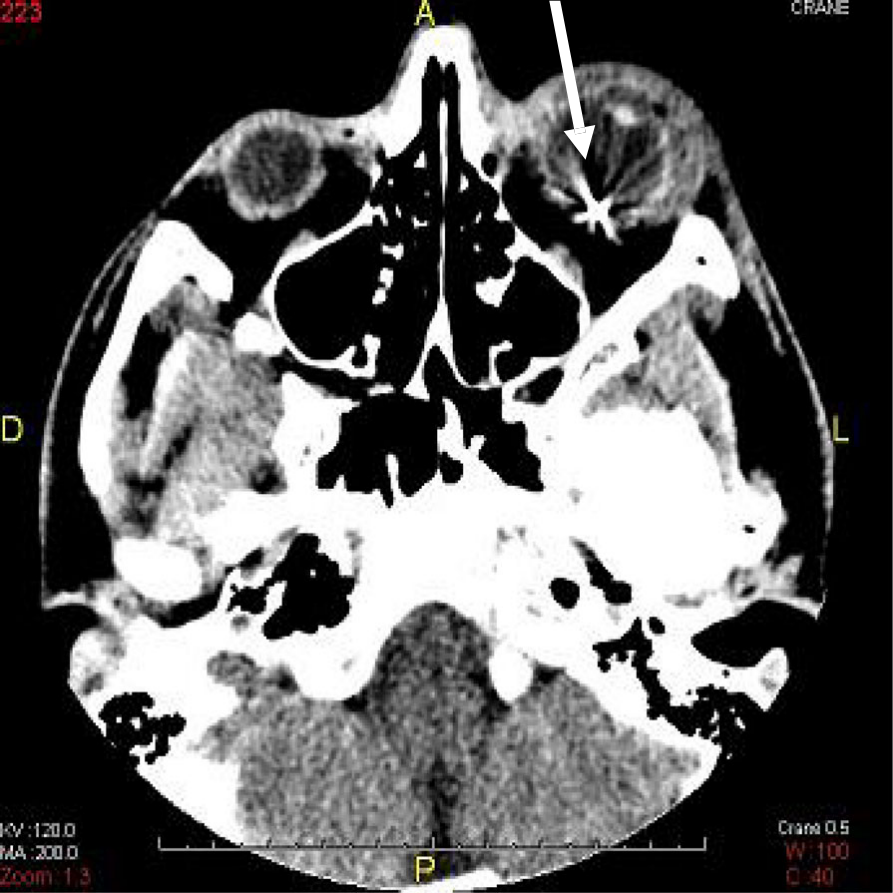
Orbital computed tomography demonstrates a metallic intraocular foreign body with edema of the left eye

**Fig. 3:**
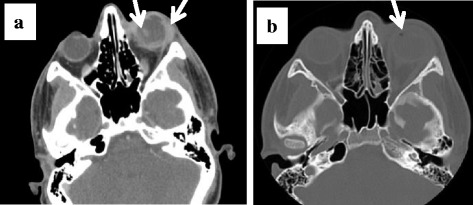
**a** Orbital computed tomography showing proptosis, posterior dislocation of the lens, thickening of the periorbital soft tissue and the posterior wall of the globe. **b** Gas bubbles in the left eye

Orbital computed tomography (CT) showed an IOFB in the left eye [Fig. 2]. A gas bubble was visualized, associated with periseptal and retroseptal soft tissue edema [Fig. 3a-b].

Examination under general anesthesia and primary repair were performed in the emergency room. Orbital cellulitis was diagnosed. The anterior chamber was full of a gelatinous substance, which precluded vitrectomy. A 3.2 mm clear corneal incision was performed and anterior chamber washout. The IOFB was removed with a magnet.

Samples of discharge from the site of injury, anterior chamber aspirate and vitreous tap were sent to the Clinical Bacteriology Department for examination. Intravitreal antibiotics were then administered (ceftazidime 2.25 mg/0.1 mL and vancomycin 1 mg/0.1 mL) following surgery.

Examination on postoperative day 1 found a slightly hypotonic globe; chemosis with considerable redness, and corneal infiltrate, while the fundus could still not be visualized. A diagnosis of panophthalmitis with lens expulsion was proposed [Fig. 3a].

B-scan ultrasound showed dense vitreous opacities with choroidal thickening [Fig. 3a]. Visual acuity remained NLP.

Gram stain of the corneal specimen in the Clinical Bacteriology Department revealed a large number of white blood cells and numerous gram-positive bacilli. On postoperative day 2, all culture samples yielded *C. perfringens,* which was identified by Matrix-Assisted Laser Desorption-Ionization Time-of-Light Mass Spectrometry with a very good validity score of > 2 (2.2). The isolate was sensitive to a wide range of the antibiotics usually used for anaerobic bacteria.

On May 31, systemic imipenem 50 mg/kg every 6 h, metronidazole 500 mg 4 times daily and gentamicin 6 mg/ kg were then administered simultaneously. Anticoagulants were added to prevent septic thrombophlebitis. Gentamicin (8 μg) and ceftazidime were injected into the subconjunctival space daily.

Follow-up CT scan revealed orbital cellulitis with no cerebral thrombophlebitis or abscess. Despite treatment, the patient’s vision remained NLP with total hypopyon and no evidence of resolution of the infection was observed at this time. Loss of vision was rapid and complete with a flat electroretinogram of the left eye.

On June 3, the anterior chamber was washed out under topical anesthesia, but the thick cyclitic membrane could not be completely removed. Intravitreal ceftazidime and vancomycin were also administered simultaneously. Ceftazidime, vancomycin and gentamicin eye drops were administered postoperatively.

On June 4, inflammation had significantly decreased in response to intensive antibiotic therapy and topical steroids. The white blood cell count and CRP levels had decreased to 9700 cells/mm^3^ and 6 mg/L, respectively.

The patient continued to improve and was discharged from hospital on June 11 with oral amoxicillin 2 g, 3 times daily and metronidazole 500 mg, 3 times daily for 10 days. Evisceration was considered due to the loss of vision, underlying infection and inflammation. However, the patient declined this procedure and the infection was completely eradicated. Evisceration was therefore not performed.

**Fig. 4:**
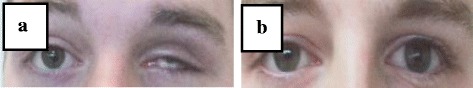
**a** Phthisis bulbi, (**b**) Esthetic overlay prosthesis

An esthetic overlay prosthesis was performed 4 weeks later due to the phthisis bulbi caused by loss of consistency of the internal structures of the eye, [Fig. 4].

## Discussion

Post-traumatic endophthalmitis can be due to both Gram-positive and Gram-negative micro-organisms. Polymicrobial and fungal infections also have been reported. Gram-positive organisms such as *Staphylococcus epidermidis* and *Bacillus cereus* are the organisms most commonly cultured [1]. *C. perfringens* is an uncommon cause of endophthalmitis and typically occurs after penetrating injury by soil- and plant-contaminated foreign bodies, causing rapid destruction of ocular tissues [6]. However, despite the potential frequency of contamination, intraocular *C. perfringens* infection is rare.

*C. perfringens* is a spore-forming anaerobic exotoxin-producing gram-positive bacillus present ubiquitously in the environment. On blood agar, *C. perfringens* produces a double zone of hemolysis due to the alpha and beta toxins produced by this species. The ocular destruction caused by *C. perfringens* infection is related to massive necrosis of ocular structures by potent exotoxins. Antibiotics are therefore unlikely to prevent this process once the infection has become established.

Traumatic intraocular inoculation of *C. perfringens* can result in fulminant endophthalmitis with little hope of a positive outcome.

Clinical signs of infection associated with this pathogen include gas bubbles in the anterior chamber, amaurosis, and greenish-brown hypopyon. These signs usually occur rapidly, often within 24 h after injury.

As described above, *C. perfringens* panophthalmitis is a devastating malignant eye infection because the bacteria produce a multitude of extracellular tissue-destructive factors. The case reported here presented no signs of systemic dissemination and the toxins were confined to the globe.

All previously reported cases progressed despite aggressive treatment, often resulting in evisceration or enucleation [5, 7]. In the rare cases in which the eye was preserved, the visual outcome remained poor.

The case reported here presented the classic clinical signs of clostridial endophthalmitis. Despite aggressive treatment including intravitreal and systemic antibiotics, the patient had a poor visual outcome, but without evisceration. Systemic antibiotics were used in combination with intravitreal antibiotics. The antibiotics most commonly used for post-traumatic endophthalmitis are vancomycin and ceftazidime [1]. Topical antibiotics are almost always used together with intravitreal antibiotics for the treatment of endophthalmitis in an attempt to increase intraocular antibiotic concentrations.

The efficacy of vitrectomy in *C. perfringens* endophthalmitis has been previously reported [8]. Vitrectomy can remove a considerable proportion of the infectious inoculum, clear the media by removing vitreous membranes and debris, and eliminate vitreous scaffolding that may cause tractional retinal detachment.

Vitrectomy combined with intravitreal antibiotics may be a more effective strategy to sterilize the vitreous cavity compared to intravitreal antibiotics alone [1, 9–11]. When visualization is compromised, endoscopy-guided vitrectomy may be the best treatment option in these difficult cases.

## Conclusion

In conclusion, this case report highlights the fact that, due to the rapidly devastating outcome, penetrating eye injuries with soil-contaminated foreign bodies must be considered to be at high risk for clostridial infection and should be treated promptly by vitrectomy and antibiotics. Otherwise, delayed surgery results in evisceration or poor visual outcomes.

Early vitrectomy and early removal of foreign material from the eye is important because this removes the growth media and potential source of infection. Vitrectomy also reduces the inoculum of harmful pathogens.

## Abbreviations

CRP: C-reactive protein
CT: Computed tomography
IOFB: Intraocular foreign body
MALDI-TOF MS: Matrix-Assisted Laser Desorption-Ionization Time-of-Light Mass Spectrometry
NLP: No light perception
